# Effects of lanreotide Autogel primary therapy on symptoms and quality-of-life in acromegaly: data from the PRIMARYS study

**DOI:** 10.1007/s11102-015-0693-y

**Published:** 2015-11-24

**Authors:** Philippe J. Caron, John S. Bevan, Stephan Petersenn, Aude Houchard, Caroline Sert, Susan M. Webb, The PRIMARYS Investigators Group

**Affiliations:** Department of Endocrinology and Metabolic Diseases, Centre Hospitalier Universitaire Larrey, Toulouse, France; Department of Endocrinology, Aberdeen Royal Infirmary, Aberdeen, UK; ENDOC Center for Endocrine Tumors, Hamburg, Germany; Ipsen, Boulogne-Billancourt, France; Departments of Endocrinology/Medicine, Sant Pau Biomedical Research Institute, Hospital de Sant Pau, Universitat Autònoma de Barcelona, Barcelona, Spain; Center for Biomedical Network Research on Rare Diseases (CIBERER Unit 747), ISCIII, Barcelona, Spain

**Keywords:** Acromegaly, Lanreotide Autogel, Quality of life, Symptoms

## Abstract

**Purpose:**

To evaluate the effects of lanreotide Autogel on patient-reported outcomes and association with biochemical control, using PRIMARYS data.

**Methods:**

PRIMARYS was a 1-year, open-label study of lanreotide Autogel (Depot in USA) 120 mg every 4 weeks in 90 treatment-naïve patients with acromegaly. Symptoms were assessed using Patient-assessed Acromegaly Symptom Questionnaire (PASQ) and health-related quality of life (HRQoL) using the AcroQoL questionnaire. Correlations between PASQ and AcroQoL scores, and between PASQ/AcroQoL and growth hormone (GH)/insulin-like growth factor-1 (IGF-1) levels were also evaluated (post hoc).

**Results:**

Acromegaly symptoms and HRQoL significantly improved from week 12 to week 48, with modest correlations at week 48 between PASQ total score (R = –0.55, *p* < 0.0001) and AcroQoL global and physical scores (R = –0.67, *p* < 0.0001). Approximately 60 % of patients achieved a minimal important difference (MID; improvement >50 % of baseline standard deviation) in PASQ total score and >40 % achieved a MID in AcroQoL global score (post hoc). Changes in PASQ scores were similar in biochemically controlled (GH levels ≤2.5 μg/L and normal IGF-1 levels) and uncontrolled groups, while changes in global and psychological AcroQoL scores were greater in the controlled group. There was no correlation between changes in PASQ or AcroQoL scores and changes in GH or IGF-1 levels.

**Conclusions:**

Primary treatment with lanreotide Autogel over 1 year was associated with rapid and sustained improvements in clinical signs and symptoms and HRQoL in patients with acromegaly. Improvements in HRQoL, but not symptoms, were greater in those achieving biochemical control (ClinicalTrials.gov: NCT00690898; EudraCT: 2007–000155–34).

**Electronic supplementary material:**

The online version of this article (doi:10.1007/s11102-015-0693-y) contains supplementary material, which is available to authorized users.

## Introduction

Acromegaly is a multisystem disease, characterized by somatic overgrowth, multiple comorbidities (including diabetes and cardiovascular disorders), premature mortality, and physical disfigurement [1, 2]. Perhaps not surprisingly, acromegaly is associated with psychological issues such as mood swings, impaired self-esteem, and disruptions in interpersonal relationships [2]. Furthermore, studies have shown that patients with acromegaly have impaired health-related quality of life (HRQoL), measured using both generic and disease-specific (AcroQoL) questionnaires [3, 4].

Treatment options for acromegaly include surgery, drug treatment, and radiotherapy. Goals of treatment are amelioration of clinical signs and symptoms, reduction of morbidity and mortality, and control of GH and IGF-1 hypersecretion and tumor growth [1, 5, 6]. The somatostatin analogs (SSAs), which include lanreotide Autogel (Depot in the USA), are largely recognized for their role in patients whose disease persists despite surgical intervention [1, 5, 6]. In addition, a number of guidelines on the management of acromegaly advocate the first-line use of long-acting SSAs in those who cannot be cured using surgery or who are poor surgical candidates [1, 5, 6]. SSAs have been shown to improve acromegaly symptoms and limit GH/IGF-1 hypersecretion and tumor growth [7–10]. There is also evidence that SSAs improve HRQoL in acromegaly [11–13].

PRIMARYS was a robust, 1-year study designed to evaluate the effect of primary treatment with lanreotide Autogel 120 mg every 4 weeks in 90 treatment-naïve patients with acromegaly [7]. The results showed that clinically significant tumor shrinkage (≥20 %) was achieved in 63 % of patients and there were early and sustained improvements in growth hormone (GH) and insulin-like growth factor-1 (IGF-1) levels, acromegaly symptoms, and HRQoL [7]. Lanreotide Autogel was well tolerated, with no patients discontinuing treatment due to gastrointestinal intolerance. The patient population enrolled in PRIMARYS was particularly homogeneous: all patients were treatment naïve and all were treated first-line with the highest available dose of lanreotide Autogel from the outset (i.e. there was no dose titration) in order to achieve rapid efficacy. In view of this, the relatively large number of patients enrolled, and the long duration of follow-up, we conducted a more in-depth analysis to further investigate the effects of treatment on patient-reported outcomes.

## Methods

The methods used for this trial (ClinicalTrials.gov: NCT00690898 and EudraCT: 2007–000155–34) have previously been described in detail [7].

### Patients

The study included treatment-naïve men and women, aged 18–75 years, diagnosed with acromegaly, with mean GH levels >1 µg/L and IGF-1 levels above the normal age- and sex-matched range. All patients had a GH-secreting macroadenoma (diameter ≥10 mm) without visual field defects. Patients were excluded if they had: undergone or were likely to need pituitary surgery or radiotherapy; previously received treatment with a SSA, dopamine agonist, or GH receptor antagonist; prolactin co-secretion >100 µg/L; optic nerve disease or any visual abnormality that may have worsened during the trial; or a glomerular filtration rate <30 mL/min/1.73 m^2^.

Patients could be withdrawn from the study if they had an insufficient IGF-1 response (<10 % reduction in IGF–1 at Week 24 or an inadequate response in the investigator’s opinion), if there was suspicion of visual field deterioration, in the event of a post-baseline prolactin level >100 µg/L, or if there were any other safety concerns.

### Trial design and interventions

This was an international, 48-week, open-label, single-arm phase 3b trial, conducted in 27 specialist endocrine centers in nine countries (Belgium, Czech Republic, Finland, France, Germany, Italy, The Netherlands, Turkey, and the United Kingdom) between May 20, 2008 and February 13, 2012. Patients received twelve injections of lanreotide Autogel 120 mg by deep subcutaneous injection every 28 days. Dose titration was not permitted.

All patients gave written informed consent to participate before the start of the trial, which was conducted in accordance with the Declaration of Helsinki, Good Clinical Practice guidelines, and all local regulatory requirements. Trial documentation was approved by institutional review boards before the start of the trial.

### Assessments and endpoints

Trial visits were conducted at baseline (week 1) and at weeks 12, 24, and 48. GH and IGF-1 levels were measured at each trial visit, as described previously [7]. The primary endpoint (change from baseline in pituitary tumor volume at week 48) and all secondary endpoints of the trial have been reported previously; here we present further detailed analyses on changes in acromegaly symptoms and changes in HRQoL. Symptoms were assessed at each visit using the Patient-assessed Acromegaly Symptom Questionnaire (PASQ), which allows patients to rate five key symptoms (headache, excessive perspiration, fatigue, soft tissue swelling, arthralgia) on a 9-point scale (0, no symptoms; 8, severe incapacitating symptoms) [14]. The total PASQ score is the sum of the individual symptom scores (maximum = 40). HRQoL was assessed at each visit using the AcroQoL questionnaire (except Finland and Turkey, where validated translations were not available). The AcroQoL questionnaire is a reliable, validated tool [3] that assesses global HRQoL, physical performance, and psychological wellbeing. The psychological wellbeing dimension is further divided into sub-dimensions for ‘appearance’ and ‘personal relationships’. Scores were standardized from 0 to 100, with higher scores in all cases representing better HRQoL.

### Statistical analyses

Summary statistics were based on the intention-to-treat (ITT) population and calculated for PASQ and AcroQoL scores at baseline, weeks 12, 24, and 48, and last post-baseline value available (LVA), and for the change in scores from baseline at these timepoints. Post hoc analyses were conducted to determine the percentage of patients with minimally important differences (MIDs) in PASQ and AcroQoL scores, defined as a change (improvement) >50 % of the baseline standard deviation (SD) of each score [15]. Post hoc analyses were also conducted to evaluate the following correlations in the overall population: total PASQ score versus AcroQoL global, physical and psychological dimension scores (Pearson correlation); individual PASQ symptom scores versus AcroQoL global, physical and psychological dimension scores (Kendall correlation); total PASQ score versus IGF-1 and GH levels, and AcroQoL global and subscale scores versus IGF-1 and GH levels. In all cases, correlations were conducted for absolute scores at week 48 and for changes in scores from baseline to week 48. Finally, post hoc analyses were conducted to evaluate baseline characteristics, total PASQ score, and global and physical AcroQoL dimension scores in patients with and without biochemical control, defined as GH levels ≤2.5 μg/L and normal IGF-1 levels, at LVA.

## Results

### Patient disposition and baseline characteristics

Ninety patients received treatment and 89 were included in the ITT population; 26 patients (29 %) withdrew before week 48 and 64 patients (71 %) completed the trial. The most common reason for withdrawal was insufficient IGF-1 response as per protocol at week 12 (n = 18), followed by consent withdrawn (n = 4), adverse events (n = 3), and other reasons (n = 1). One patient in the ITT population did not have data available on biochemical control; for this reason, there were 88 patients in the overall population in the current analyses. Of these, all completed the PASQ at baseline and 83 completed the AcroQoL questionnaire.

Baseline demography and clinical characteristics are summarized in Table 1 for the overall population and for those with and without biochemical control, and in Supplementary Table S1 for the 18 patients who withdrew because of insufficient IGF-1 response. Patients with biochemical control (GH ≤2.5 µg/L and IGF-1 < ULN) at LVA were significantly older at baseline than those in the uncontrolled group at LVA, and had significantly lower baseline IGF-1 levels (Table 1). Patients with biochemical control tended to be female (70 vs. 45 % in the uncontrolled group) and tended to have lower median baseline tumor volumes (1339 vs. 1786 mm^3^ in the uncontrolled group). There were no statistically or clinically significant differences in baseline characteristics between the group who withdrew because of insufficient response and the ITT population excluding these 18 patients (Table 1).

**Table 1:** Baseline demography and disease characteristics in the overall population and according to achievement of biochemical control

	Overall population (n = 88)^a^	Patients achieving biochemical control (n = 30)	Patients not achieving biochemical control (n = 58)
Age, years	49.5 (46.9, 52.1)	*56.1 (52.4, 59.7)*	*46.1 (42.8, 49.3)*
Female, n	47	21	26
% (95 % CI)	53.4 (42.5, 64.1)	70.0 (50.6, 85.3)	44.8 (31.7, 58.5)
BMI, kg/m^2^	27.7 (26.7, 28.7)	26.4 (24.9, 27.9)	28.4 (27.1, 29.7)
Time since acromegaly diagnosis, days	121.1 (84.6, 157.6)	147.0 (44.8, 249.3)	107.3 (87.1, 127.5)
Maximum tumor diameter, mm			
Mean (95 % CI)	18.9 (17.4, 20.4)	17.3 (15.6, 19.1)	19.8 (17.7, 21.9)
Median (interquartile range)	18.1 (13.9, 21.8)	17.2 (13.4, 20.2)	18.2 (14.2, 23.0)
Maximum tumor volume, mm^3^			
Mean (95 % CI)	2731 (2033, 3428)	1822 (1211, 2433)	3201 (2199, 4203)
Median (interquartile range)	1653 (866, 3370)	1339 (811, 2325)	1786 (958, 4404)
GH level, µg/L			
Mean (95 % CI)	15.2 (11.2, 19.2)	13.0 (6.6, 19.3)	16.3 (11.1, 21.6)
Median (interquartile range)	8.6 (3.8, 16.8)	6.4 (2.9, 14.9)	9.7 (4.6, 17.3)
IGF-1 level, µg/L			
Mean (95 % CI)	796 (735, 857)	*665 (563, 767)*	*863 (792, 935)*
Median (interquartile range)	778 (594, 973)	*630 (472, 794)*	*805 (674, 1010)*
IGF-1 levels, % of ULN	285 (260.6, 309.3)	249 (211.2, 286.7)	304 (272.6, 334.6)
PASQ scores, mean (95 % CI)			
Total	19.4 (17.7, 21.1)	19.8 (16.9, 22.7)	19.2 (17.0, 21.5)
Headache	2.8 (2.2, 3.3)	2.0 (1.2, 2.9)	3.1 (2.5, 3.8)
Excessive perspiration	4.0 (3.4, 4.5)	4.1 (3.2, 5.1)	3.9 (3.2, 4.6)
Fatigue	4.5 (4.0, 5.0)	5.0 (4.1, 5.9)	4.3 (3.7, 4.9)
Soft tissue swelling	4.3 (3.7, 4.8)	4.6 (3.7, 5.5)	4.1 (3.5, 4.7)
Arthralgia	3.9 (3.4, 4.5)	4.1 (3.2, 5.0)	3.8 (3.1, 4.6)
AcroQoL scores^b^, mean (95 % CI)			
Global	56.0 (52.5, 59.5)	50.9 (45.1, 56.7)	58.8 (54.4, 63.1)
Physical	54.6 (50.4, 58.8)	52.2 (44.6, 59.7)	55.9 (50.7, 61.1)
Psychological	56.9 (53.1, 60.6)	50.2 (44.5, 56.0)	60.5 (55.7, 65.2)
Psychological (appearance)	41.4 (37.0, 45.7)	34.1 (27.8, 40.4)	45.4 (39.7, 51.0)
Psychological (personal relationships)	71.7 (67.6, 75.7)	65.1 (58.0, 72.2)	75.0 (70.1, 79.8)

*CI* confidence interval, *BMI* body mass index, *IGF-1* insulin-like growth factor-1, *GH* growth hormone, *SD* standard deviation, *ULN* upper limit of normal

^a^Excludes one patient in the ITT population with no data available on biochemical control. Data are the mean (95 % CI) unless otherwise specified; italic cells indicate parameters with non-overlapping CIs for biochemical control and lack of biochemical control (i.e. statistically significant differences). Biochemical control defined as GH levels ≤2.5 μg/L and normal IGF-1 levels at last post-baseline value available

^b^n = 83 for overall population (29 for controlled, 54 for uncontrolled)

### IGF-1, acromegaly symptoms, and HRQoL in the overall population

As previously reported (7), mean IGF-1 levels were greatly reduced at week 12 versus baseline, and reductions were maintained until the end of the trial.

**Fig. 1:**
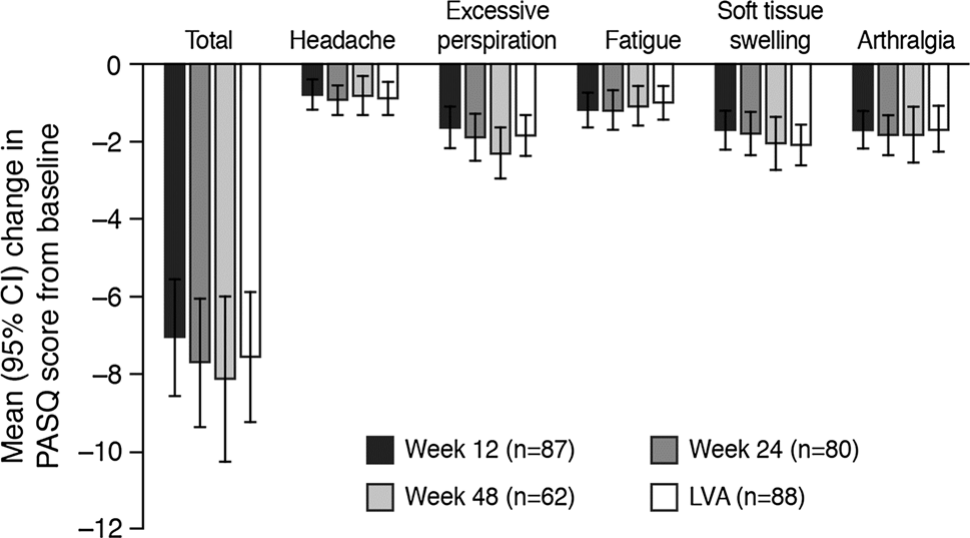
Mean (95 % CI) change in PASQ scores during treatment with Lanreotide Autogel. Each symptom is assessed on a 9-point scale, total maximum score = 40 (higher scores indicate worse symptoms); baseline scores are summarized in Table 1. Full data for this Figure are provided in Supplementary Table S2. *CI* confidence interval, *PASQ* patient-assessed acromegaly symptom questionnaire

PASQ scores at baseline (n = 88) and during treatment (week 12, n = 87; week 48, n = 62) are shown in Table 1 and Fig. 1, and the percentage of patients with a MID in PASQ is shown in Supplementary Figure S1. The most troublesome symptoms at baseline were fatigue and soft tissue swelling, whereas headache was least troublesome (Table 1). The PASQ total score decreased significantly between baseline and week 12 (mean change, −7.1); this decrease was maintained at LVA (mean change, −7.6) (Fig. 1). Early and sustained reductions were also observed in each of the individual symptom scores. At LVA, greatest mean change was in excessive perspiration (−1.9) and smallest mean change was in headache (−0.9). Over half of patients experienced a MID in their PASQ total score at week 12 (59 %) and LVA (63 %). For the individual symptoms, excessive perspiration and soft tissue swelling showed greatest improvement, with MIDs in 51 %/51 % (week 12/LVA) and 49 %/62 % of patients, respectively, while the lowest MID rates were for headache with 26 %/30 % (Figure S1).

**Fig. 2:**
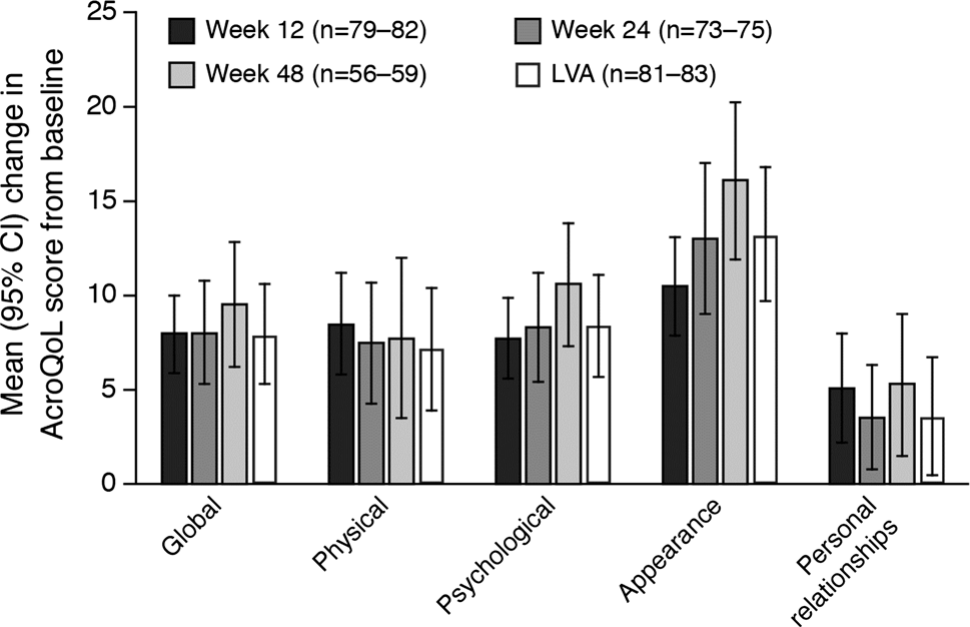
Mean (95 % CI) change in AcroQoL scores during treatment with Lanreotide Autogel. Scores for each dimension/sub-dimension are standardized from 0 to 100 (higher scores = better HRQoL); baseline scores are summarized in Table 1. Full data for this Figure are provided in Supplementary Table S2. *CI* confidence interval, *AcroQoL* acromegaly quality of life questionnaire

AcroQoL scores at baseline (n = 81–83) and during the study (week 12, n = 81–82; week 48, n = 57–59) are shown in Table 1 and Fig. 2, and the percentage of patients with a MID during treatment is shown in Supplementary Figure S1. At baseline, the greatest impairment was for the psychological sub-dimension, appearance and the least impairment was for the psychological sub-dimension, personal relationships (Table 1). The improvement in the global AcroQoL score from baseline was significant at week 12 (mean change, 8.0) and at LVA (mean change, 7.9) (Fig. 2). During treatment, the greatest improvements in each of the dimension or sub-dimensions were seen between baseline and week 12; these improvements continued (psychological dimension, appearance sub-dimension) or remained relatively stable (physical dimension, personal relationships sub-dimension) through to week 48. MID rates during treatment for the AcroQoL global score were 43 % at week 12 and 42 % at LVA, with appearance showing the greatest improvement (52 %/55 %), and personal relationships sub-dimension showing the least improvement (33 %/28 %) (Figure S1).

### Correlation analyses in the overall population

For week-48 scores, there was a moderate correlation between the total PASQ and both global and physical AcroQoL scores (R = −0.55 and R = −0.67; both *p* < 0.0001), but not psychological AcroQoL dimension scores (R = −0.27, *p* = 0.0017; Supplementary Table S3). The correlations between the total PASQ score and AcroQoL domain scores were stronger than those between the individual PASQ symptom scores and the AcroQoL domain scores. For changes from baseline to week-48 scores, there were no correlations between PASQ total/individual symptom scores and AcroQoL domains. Finally, there were no correlations for week-48 scores or changes from baseline to week 48 for GH/IGF-1 levels and either PASQ or AcroQoL scores.

### Acromegaly symptoms and HRQoL in patients according to biochemical response

**Fig. 3:**
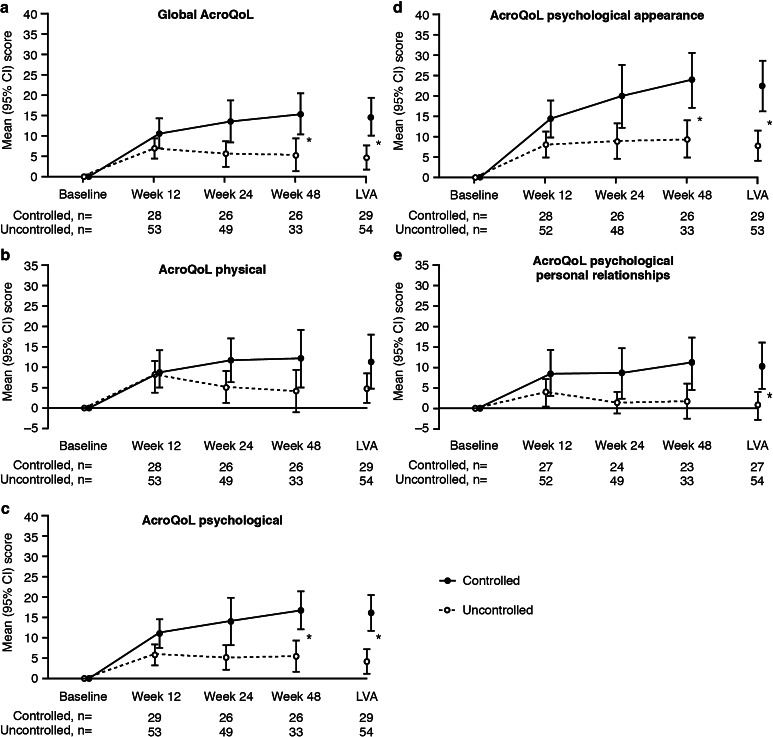
Changes in AcroQoL scores for patients with and without biochemical control during treatment with Lanreotide Autogel. *AcroQoL* acromegaly quality of life questionnaire, *CI* confidence interval. *Asterisks* indicate non-overlapping CIs, i.e. statistically significant between-group differences. Baseline scores are summarized in Table 1

At baseline, PASQ symptom scores were similar in the two groups (Table 1). In addition, there were no significant differences in the change in symptom scores from baseline to LVA between the biochemically controlled and the uncontrolled groups (Supplementary Table S4). There was no significant difference in baseline AcroQoL scores in the groups with and without subsequent biochemical control (Table 1). However, the mean [95 % CI] change in the global AcroQoL score at LVA was significantly higher in the controlled group (14.4 [9.8–19.0]) than in the uncontrolled subjects (4.4 [1.5–7.4]), as were the changes in the psychological dimension, and the appearance and personal relationships sub-dimension scores (Fig. 3). Conversely, there was no significant difference between the controlled and uncontrolled groups for the physical AcroQoL score at LVA.

Changes in PASQ total and AcroQoL global scores in the 18 patients who withdrew because of insufficient IGF-1 response are summarized in Supplementary Figure S2. Compared with the ITT population excluding these 18 patients, there appeared to be smaller improvements in PASQ and AcroQoL scores, but between-group differences were not statistically significant.

## Discussion

The results of the current analyses show that primary medical treatment of acromegaly with lanreotide Autogel 120 mg is associated with early and sustained improvements in patient-reported symptoms and HRQoL. Improvements were noted across all symptoms evaluated (headache, excessive perspiration, fatigue, soft tissue swelling, and arthralgia) and approximately 60 % of patients had a clinically significant improvement (MID) in their total symptom score at LVA. Similarly, improvements were noted across all HRQoL dimension and sub-dimension scores (physical, psychological, appearance, and personal relationships) and >40 % of patients had a clinically significant improvement in their global HRQoL score at LVA. Amongst the AcroQoL dimensions and sub-dimensions, the greatest proportion of patients had clinically significant improvements in the appearance sub-dimension. This sub-dimension also scored worst at baseline, so the greater effect of treatment may reflect a larger scope for improvement.

As reported previously [7], mean GH and IGF-1 levels were significantly reduced at week 12 versus baseline, and improvements were maintained until the end of the trial. Improvements in acromegaly symptoms were also greatest during the first 12 weeks. Interestingly, however, the total and individual symptom scores during the study did not differ in those who achieved biochemical control (GH ≤2.5 μg/L and normal IGF-1 levels), compared with those who did not. Furthermore, there were no correlations between symptom scores and GH or IGF-1 levels. These results suggest that there may be a dissociation between hormonal control and symptom improvement, possibly reflecting patients’ long-term experience of the disease before diagnosis, the effect of comorbidities, or underlying differences in baseline characteristics between those with and without biochemical control (e.g. age and possibly gender). With regards to HRQoL, there was a modest correlation between the total PASQ and both global and physical AcroQoL scores. There were no correlations between any of these HRQoL scores and GH or IGF-1 levels, but improvements in the global and psychological AcroQoL scores were significantly greater in those who achieved biochemical control compared with those who did not. A number of previous studies have evaluated the association between HRQoL and biochemical control; the results are mixed, with some showing better HRQoL in those with biochemical control, and others showing no such association [16, 17]. Interestingly, in one study, IGF-1 control was associated with better scores on the AcroQoL appearance sub-dimension, but not for the global, physical or psychological dimensions [18]. In another study, administration of pegvisomant to patients biochemically controlled with a SSA resulted in an improvement in HRQoL despite no change in IGF-1 levels [14]. The relationship between biochemical control (as determined by circulating hormones) and HRQoL therefore remains to be determined, but it should be borne in mind that HRQoL can be affected by a number of factors associated with acromegaly and the treatment modality. These include cosmetic or orthopedic deformities or comorbidities, which may already be established in some patients, particularly in view of the time lag between symptom onset and diagnosis [16]. It has also been shown that illness perception affects HRQoL in those with acromegaly [19]. In addition, tissue sensitivity to GH and IGF-1 excess can vary [14], which may also explain the apparent disconnect between HRQoL and circulating levels of GH and IGF-1 in some studies. Finally, it should be borne in mind that the studies in which HRQoL and biochemical control have been assessed include heterogeneous populations in terms of treatments received (surgery, radiotherapy, medical treatment, or a combination of these) [14, 16–18], which could also impact on HRQoL. Indeed, one of the strengths of the current study was the homogeneity of the patient population, who were all treatment naïve.

Other studies have used the PASQ and AcroQoL to assess symptoms and HRQoL in those treated for acromegaly [9, 12, 20–23]. In the observational ACROSTUDY of the GH receptor antagonist, pegvisomant, only the PASQ score for excessive perspiration was significantly improved following 1 year’s treatment; after 2 years, numbness and soft tissue swelling were significantly reduced [20]. HRQoL was assessed using AcroQoL in a recent randomized study comparing octreotide LAR and pasireotide LAR in 358 medical treatment-naïve patients; baseline scores (55.6 and 58.4) were similar to those in the current study (56.0). After 1 year, scores increased by 4.9 in the octreotide LAR group and 7.0 in the pasireotide group; the results in the current study (increase of 9.5) compare well with these [7]. Larger increases in the AcroQoL global score (+17) were noted with octreotide LAR in an earlier observational study, but it included only 28 patients treated for 4 years, so the results are more difficult to compare [12].

One limitation of the current study was its open-label, uncontrolled design. However, this was felt to be justified because of the objective nature of the primary endpoint, tumor volume reduction [7]. Strengths of the study, particularly in the context of the patient-reported outcomes, include the relatively large size of the patient population, its homogeneity, and the long duration of follow-up. Many of the previously reported studies evaluating the effect of SSAs on HRQoL included fewer than 50 patients [12–14, 24–26]. Furthermore, unlike many of the other studies [11, 12, 14, 26], patients were treatment naïve, and as such, they represent an appropriate population for evaluating patient-reported outcomes and HRQoL in the earlier stages of treatment.

The results of the current study confirmed that medical treatment with lanreotide provided meaningful improvements not only in hormone levels, but also in clinical outcomes as evaluated by the PASQ and AcroQoL questionnaires. The relationship between biochemical and PASQ improvements was not clear, whereas the association between biochemical and AcroQoL improvements was more apparent. This divergence highlights the importance, wherever practicable, of using more than one appropriate questionnaire, in addition to biochemical measures, to fully evaluate clinical outcomes during medical treatment for acromegaly. Such an approach will offer different, but complementary, information on treatment response. In particular, it is most important to consider using a tool, such as AcroQoL, that assesses patients’ subjective perceptions of the impact of disease on their daily HRQoL, as this outcome cannot always be reliably inferred from biochemical markers or objective symptom evaluation.

In conclusion, primary treatment with lanreotide Autogel 120 mg every 4 weeks for 1 year was associated with early and sustained improvements in signs and symptoms and HRQoL in patients with acromegaly. Symptom improvement did not appear to depend exclusively on biochemical control. On the other hand, improvements in HRQoL may reflect improvements in clinical signs and symptoms in these patients as well as a decrease of GH/IGF-1 hypersecretion. These results highlight the importance of using appropriate questionnaires to fully evaluate clinical outcomes during medical treatment for acromegaly.

### Electronic supplementary material

Supplementary material 1 (DOCX 370 kb)

## Appendix

### PRIMARYS Study Group

**Belgium** L. Van Gaal; **Czech Republic** J. Marek; **Finland** P. Nuutila, M. Välimäki; **France** C. Ajzenberg, F. Borson-Chazot, T. Brue, P. Caron, O. Chabre, P. Chanson, C. Cortet Rudelli, B. Delemer, J.-M. Kuhn, A. Tabarin; **Germany** K. Badenhoop, C. Berg, S. Petersenn, C. Schöfl, J. Schopohl; **Italy** S. Cannavò, A. Colao, L. De Marinis; **the Netherlands** A. Stades, A.J. van der Lely; Turkey P. Kadıoğlu; **UK** J.S. Bevan, D. Flanagan, P. Trainer.
