# Supplementary material for: Effects of lanreotide Autogel primary therapy on symptoms and quality-of-life in acromegaly: data from the PRIMARYS study
Source: Pituitary. 2015 Nov 24;19:149–57. doi: 10.1007/s11102-015-0693-y (PMC4799252; doi:10.1007/s11102-015-0693-y)
Supplement: Supplementary file 1 — Supplementary material 1 (DOCX 370 kb) [file 11102_2015_693_MOESM1_ESM.docx]

**Supplemental Figures and Tables**

**Supplementary Fig. S1.** MID rates PASQ scores and AcroQoL for Lanreotide Autogel at LVA


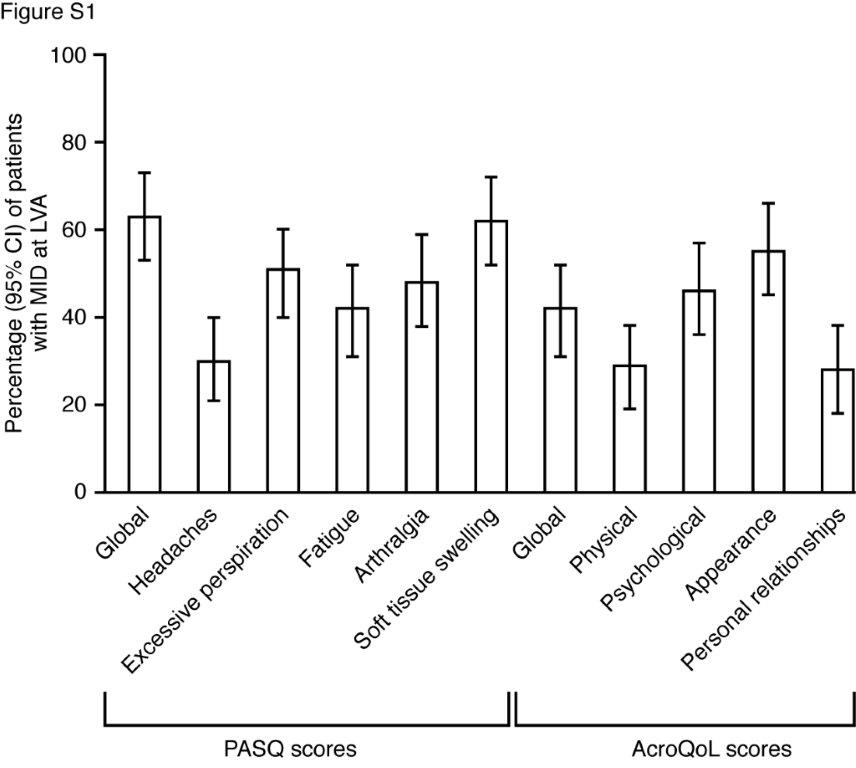


AcroQoL, Acromegaly Quality of Life Questionnaire; CI, confidence interval; MID, minimal important difference (>50% of the baseline standard deviation); LVA, last post-baseline value available; PASQ, Patient-assessed Acromegaly Symptom Questionnaire.

**Supplementary Fig. S2.** PASQ (a) and AcroQoL (b) global scores in patients who withdrew because of insufficient IGF-1 response vs. the ITT population excluding those with insufficient IGF-1 response.


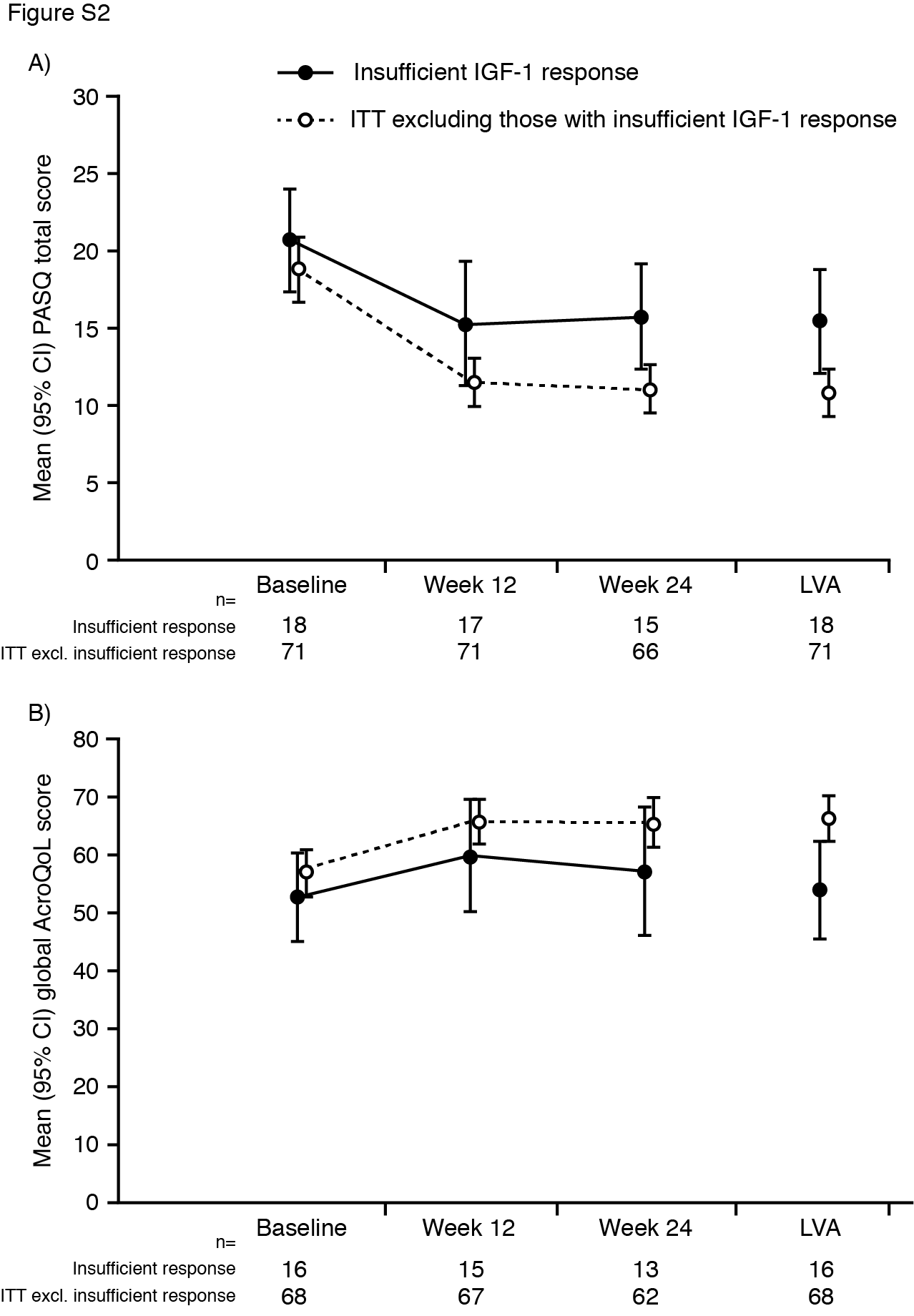


95% CIs were overlapping at all timepoints, demonstrating no significant differences between groups
AcroQoL, Acromegaly Quality of Life Questionnaire; CI, confidence interval; IGF-1, insulin-like growth factor-1; ITT, intention-to-treat; PASQ, Patient-assessed Acromegaly Symptom Questionnaire

**Supplementary Table S1.** Baseline Demography and Disease Characteristics of Patients who Withdrew because of Insufficient IGF-1 Response vs. ITT Population Excluding these Patients

|  | **Insufficient IGF-1 response**  **(n=18)** | **ITT excluding those with insufficient IGF-1 response**  **(n=71)** |
| --- | --- | --- |
| Age, y | 43.2 (35.5, 50.8) | 51.3 (48.6, 53.9) |
| Women, n (%)  [95% CI] | 9 (50)  [26, 74] | 38 (53.5)  [41, 66] |
| GH level, µg/L  Mean (95% CI)  Median (interquartile range) | 15.8 (4.9, 26.7)  8.6 (4.6, 15.2) | 14.9 (10.6, 19.2)  8.5 (3.5, 16.9) |
| IGF-1 level, µg/L  Mean (95% CI)  Median (interquartile range) | 879 (753, 1005)  846 (773, 1006) | 784 (713, 856)  723 (570, 992) |
| IGF-1 level, % of ULN  Mean (95% CI)  Median (interquartile range) | 296 (231, 360)  254 (215, 338) | 288 (259, 316)  262 (209, 347) |
| Total PASQ score | 20.8 (17.5, 24.1) | 18.9 (16.8, 21.0) |
| Global AcroQoL score | 52.7 (45.0, 60.3) | 57.0 (53.1, 61.0) |

Abbreviations: CI, confidence interval; IGF-1, insulin-like growth factor-1; ITT, intention to treat; SD, standard deviation; ULN, upper limit of normal.Data are mean (95% CI) unless otherwise stated. 95% CIs were overlapping for all parameters, demonstrating no significant differences between groups.

**Supplementary Table S2.** Mean [95% CI] changes from baseline in QoL scores

|  | **Week 12** | **Week 24** | **Week 48** | **LVA** |
| --- | --- | --- | --- | --- |
| PASQ  Global  Headache  Excessive perspiration  Fatigue  Arthralgia  Soft tissue swelling | –7.1 [–8.6, –5.5]  –0.8 [–1.2, –0.4]  –1.6 [–2.2, –1.1]  –1.2 [–1.6, –0.8]  –1.7 [–2.2, –1.2]  –1.7 [–2.2, –1.2] | –7.7 [–9.3, –6.0]  –0.9 [–1.3, –0.6]  –1.9 [–2.5, –1.3]  –1.2 [–1.7, –0.7]  –1.9 [–2.4, –1.3]  –1.8 [–2.4, –1.2] | –8.1 [–10.2, –6.0]  –0.8 [–1.3, –0.3]  –2.3 [–3.0, –1.7]  –1.1 [–1.6, –0.6]  –1.8 [–2.6, –1.1]  –2.1 [–2.7, –1.4] | –7.6 [–9.2, –5.9]  –0.9 [–1.3, –0.5]  –1.9 [–2.4, –1.3]  –1.0 [–1.4, –0.6]  –1.7 [–2.3, –1.1]  –2.1 [–2.6, –1.] |
| AcroQoL  Global  Physical  Psychological  Appearance  Personal relationships | 8.0 [5.9, 10.0]  8.5 [5.8, 11.2]  7.7 [5.6, 9.8]  10.5 [7.9, 13.1]  5.1 [2.2, 8.0] | 8.0 [5.3, 10.8]  7.5 [4.3, 10.7]  8.3 [5.4, 11.2]  13.0 [9.0, 17.0]  3.5 [0.8, 6.3] | 9.5 [6.2, 12.8]  7.7 [3.5, 12.0]  10.6 [7.3, 13.8]  16.1 [11.9, 20.2]  5.3 [1.5, 9.0] | 7.9 [5.3, 10.6]  7.2 [3.9, 10.4]  8.4 [5.7, 11.1]  13.2 [9.7, 16.8]  3.6 [0.5, 6.7] |

Data are from the intention-to-treat population. CI, confidence interval; LVA, last visit available.

**Supplementary Table S3.** Correlation Data for Individual Symptoms and AcroQoL Global, Physical, and Psychological Dimension Scores

|  | **Correlation co-efficient^a^** | | | | | | | | | | | |
| --- | --- | --- | --- | --- | --- | --- | --- | --- | --- | --- | --- | --- |
|  | **AcroQoL Global Score** | | | | **AcroQoL Physical Score** | | | | **AcroQoL Psychological Score** | | | |
|  | **Baseline^b^** | **Week 12^c^** | **Week 24^d^** | **Week 48^e^** | **Baseline^b^** | **Week 12^c^** | **Week 24^d^** | **Week 48^e^** | **Baseline^b^** | **Week 12^c^** | **Week 24^d^** | **Week 48^e^** |
| Headache | –0.24  (p=0.0019) | –0.24 (p=0.0016) | –0.31 (p=0.0012) | –0.23 (p=0.0343) | –0.36 (p<0.0001) | –0.34 (p<0.0001) | –0.30 (p=0.0002) | –0.28 (p=0.0041) | –0.13  (p=0.1091) | –0.14  (p=0.0667) | –0.25  (p=0.0097) | –0.14  (p=0.1632) |
| Excessive perspiration | –0.05 (p=0.3586) | –0.16 (p=0.0084) | –0.22 (p=0.0026) | –0.09 (p=0.2866) | –0.07 (p=0.4422) | –0.11 (p=0.0284) | –0.21 (p=0.0026) | –0.08 (p=0.2888) | –0.03  (p=0.41) | –0.15  (p=0.0107) | –0.20  (p=0.0079) | –0.08  (p=0.3461) |
| Fatigue | –0.32 (p<0.0001) | –0.33 (p<0.0001) | –0.36 (p<0.0001) | –0.42 (p<0.0001) | –0.45 (p<0.0001) | –0.55 (p<0.0001) | –0.44 (p<0.0001) | –0.56 (p<0.0001) | –0.18  (p=0.362) | –0.20  (p=0.0062) | –0.28  (p=0.0015) | –0.30  (p=0.0008) |
| Soft tissue swelling | –0.25 (p=0.0016) | –0.42 (p<0.0001) | –0.24 (p=0.0122) | –0.35 (p=0.0004) | –0.28 (p=0.0006) | –0.42 (p<0.0001) | –0.27 (p=0.0077) | –0.38 (p=0.0001) | –0.20  (p=0.0167) | –0.35  (p<0.0001) | –0.21  (p=0.0332) | –0.27  (p=0.0043) |
| Arthralgia | –0.35 (p<0.0001) | –0.34 (p<0.0001) | –0.26 (p=0.0011) | –0.22 (p=0.0240) | –0.40 (p<0.0001) | –0.43 (p<0.0001) | –0.38 (p<0.0001) | –0.33 (p=0.0005) | –0.26  (p=0.0013) | –0.24  (p=0.0008) | –0.17  (p=0.0451) | –0.12  (p=0.207) |
| Global PASQ | –0.49 (p<0.0001) | –0.63 (p<0.0001) | –0.57 (p<0.0001) | –0.55 (p<0.0001) | –0.61 (p<0.0001) | –0.73 (p<0.0001) | –0.67 (p<0.0001) | –0.67 (p<0.0001) | –0.24  (p=0.0023) | –0.25  (p<0.0001) | –0.32  (p<0.0001) | –0.27  (p=0.0017) |

Data are from the intention-to-treat population.

Shaded cells represent those correlations with moderate or high correlation coefficients (R>0.6) that are considered to be clinically relevant (Hinkle DE, Wiersma W, Jurs SG. Applied Statistics for the Behavioral Sciences. 5th ed. Boston: Houghton Mifflin; 2003.)

^a^Pearson correlation co-efficient for global symptom scores, Kendall correlation co-efficient for individual symptom scores; ^b^Baseline symptom score vs. baseline AcroQoL score; ^c^Week 12 symptom score vs. week 12 AcroQoL score; ^d^Week 24 symptom score vs. week 24 AcroQoL score; ^e^Week 48 symptom score vs. week 48 AcroQoL score

**Supplementary Table S4.** Changes in GH and IGF-1 Levels and PASQ Scores According to Achievement of Biochemical Control at LVA.

|  | **Patients achieving biochemical control^b^ (n=30)** | **Patients not achieving biochemical control (n=58)** |
| --- | --- | --- |
| GH changes, µg/L  Baseline  LVA  Change | 13.0 (6.6, 19.3)  0.8 (0.6, 1.0)  –12.1 (–18.4, –5.8) | 16.3 (11.1, 21.6)  4.6 (3.3, 5.9)  –11.8 (–16.8, –6.7) |
| IGF-1 changes, µg/L  Baseline  LVA  Change | 665 (563, 767)  185 (166, 203)  –480 (–573, –387) | 863 (792, 935)  554 (488, 620)  –309 (–382, –236) |
| IGF-1 level, % of ULN  Baseline  LVA  Change | 249 (211, 287)  69 (63, 75)  –180 (–215, –145) | 159 (134, 184)  191 (169, 213)  –112 (–141, –83) |
| Headache  Baseline score  LVA score  Change ^a^ | 2.03 (1.19, 2.87)  1.10 (0.52, 1.68)  –0.93 (–1.60, –0.27) | 3.12 (2.45, 3.79)  2.26 (1.66, 2.86)  –0.86 (–1.42, –0.31) |
| Excessive perspiration  Baseline score  LVA score  Change^a^ | 4.13 (3.16, 5.11)  1.63 (0.85, 2.42)  –2.50 (–3.46, –1.54) | 3.88 (3.20, 4.56)  2.34 (1.86, 2.83)  –1.53 (–2.16, –0.91) |
| Fatigue  Baseline score  LVA score  Change ^a^ | 4.97 (4.05, 5.88)  3.77 (2.93, 4.60)  –1.20 (–1.87, –0.53) | 4.29 (3.65, 4.93)  3.40 (2.85, 3.94)  –0.90 (–1.44, –0.35) |
| Soft tissue swelling  Baseline score  LVA score  Change ^a^ | 4.57 (3.67, 5.46)  1.80 (1.11, 2.49)  –2.77 (–3.54, –2.00) | 4.09 (3.45, 4.72)  2.33 (1.86, 2.80)  –1.76 (–2.46, –1.57) |
| Arthralgia  Baseline score  LVA score  Change ^a^ | 4.07 (3.15, 4.98)  2.33 (1.39, 3.28)  –1.73 (–2.73, –0.74) | 3.84 (3.13, 4.56)  2.17 (1.63, 2.72)  –1.67 (–2.43, –0.91) |
| Total symptom score  Baseline  LVA score  Change ^a^ | 19.77 (16.85, 22.68)  10.63 (8.16, 13.11)  –9.13 (–11.75, –6.52) | 19.22 (16.96, 21.49)  12.50 (10.73, 14.27)  –6.72 (–8.89, –4.56) |

Abbreviations: LVA, last post-baseline value available; SD, standard deviation. Data are from the intention-to-treat population represented as mean (95% CI). 95% CIs were overlapping for all scores, demonstrating no significant differences between groups. ^a^Larger decrease=greater improvement; ^b^Biochemical control defined as GH levels ≤2.5 μg/L and normal IGF-1 levels at LVA.

### PRIMARYS Study Group

**Belgium** L. Van Gaal; **Czech Republic** J. Marek; **Finland** P. Nuutila, M. Välimäki; **France** C. Ajzenberg, F. Borson-Chazot, T. Brue, P. Caron, O. Chabre, P. Chanson, C. Cortet Rudelli, B. Delemer, J.-M. Kuhn, A. Tabarin; **Germany** K. Badenhoop, C. Berg, S. Petersenn, C. Schöfl , J. Schopohl; **Italy** S. Cannavò, A. Colao, L. De Marinis; **the Netherlands** A. Stades, A.J. van der Lely; Turkey P. Kadıoğlu; **UK** J.S. Bevan, D. Flanagan, P. Trainer.
